# Mitochondrial Creatine Kinase is Decreased in the Serum of Idiopathic Parkinson’s Disease Patients

**DOI:** 10.14336/AD.2018.0615

**Published:** 2019-06-01

**Authors:** Jinghui Xu, Xiaodi Fu, Mengqiu Pan, Xiao Zhou, Zhaoyu Chen, Dongmei Wang, Xiaomei Zhang, Qiong Chen, Yanhui Li, Xiaoxian Huang, Guanghui Liu, Jianjun Lu, Yan Liu, Yafang Hu, Suyue Pan, Qing Wang, Qun Wang, Yunqi Xu

**Affiliations:** ^1^Department of Neurology, Nanfang Hospital, Southern Medical University, Guangzhou, China; ^2^Department of Neurology, Guangdong 999 Brain Hospital, Guangzhou, China; ^3^Department of Neurology, the Third Affiliated Hospital, Sun Yat-sen University, Guangzhou, China; ^4^Department of Medical Imaging, Nanfang Hospital, Southern Medical University, Guangzhou, China

**Keywords:** Parkinson’s disease, mitochondrial creatine kinase, ubiquitous mitochondrial creatine kinase, sarcomeric mitochondrial creatine kinase, mitochondrial dysfunction

## Abstract

Mitochondrial creatine kinase (MtCK) is vital in the process of mitochondrial energy metabolism, and mitochondrial dysfunction has been implicated in the pathogenesis of Parkinson’s disease (PD). Therefore, we speculated that MtCK activity could be altered in the serum of PD patients. However, no studies to date have investigated this specific topic, so we sought to investigate the serum MtCK activities among a cohort of PD patients. 50 patients with PD and 30 age-matched controls were recruited for this study. Serum ubiquitous MtCK (uMtCK) and sarcomeric MtCK (sMtCK) activities were assayed using an immunoinhibition method. Correlations between serum uMtCK/sMtCK activities and clinical features/parameters were explored in the PD group. Our study revealed a significant decrease in the uMtCK activity in the PD group when compared with the control group. No significant difference was found in the serum sMtCK activity between the PD and control groups. There was a significant correlation between serum uMtCK activities and the disease progression rate, duration, and age at onset in PD patients. While no significant relationship was found between the serum uMtCK activities and the Hoehn & Yahr stage or main non-motor symptoms scale. There was a significant decrease in the uMtCK activity in the serum of PD patients, which was associated with the rate of disease progression, duration, and age at onset of disease. Therefore, uMtCK activity in serum offers a useful clue for identification of PD biomarkers.

Despite the efforts of past research, definitive evidence regarding the etiology of idiopathic Parkinson’s disease (PD) is still lacking. Some etiopathogeneses have been implicated in the development of idiopathic PD, including calcium overload, excitatory amino acid toxicity, oxidative stress, and iron deposition in the brain [1]. Previous studies have confirmed the role of mitochondrial dysfunction in the pathogenesis of PD [2-4], however, no effective drugs could be used to block disease progression, and it became obvious that the understanding of this contribution to PD pathogenesis was incomplete. Given this, there is a profoundly important need for biomarkers that reflect the pathogenetic process of PD in order to improve the ability to diagnose and predict disease outcome in patients. This is a challenging task because there are currently no known effective biomarkers that are easily obtained from the clinical population. Although many potential clinical, biochemical, imaging, and pathological biomarkers have been studied, Schapira believed that the search for an appropriate biomarker for Parkinson’s disease (PD) would continue [5].

Mitochondrial creatine kinase (MtCK) is an important component of mitochondria that is structurally and functionally coupled to ATP synthasome. There are two subtypes of MtCK. One, sMtCK, is expressed primarily in striated muscle tissue, and the other, uMtCK, is expressed primarily in the brain, kidney, and other organs [6]. Two separate nuclear genes encoded sMtCK and uMtCK [7]. Immunogold electron microscopy has revealed that MtCK is localized both in the peripheral intermembrane space (IMS) and cristae space of mitochondria [6].

MtCK is crucial for the transfer of high energy phosphate from mitochondria to the cytosolic carrier, creatine [8]. Briefly, ATP regenerated by ATP synthase in mitochondria is transferred to MtCK because of its functional coupling with ATP synthase. Next, MtCK catalyzes the transfer of the phosphate group from ATP to creatine [9]. The MtCK and the CK/phosphocreatine system might play an important role in an intricate, metabolic energy transfer network in cells, linking mitochondria with myofibrils, sarcoplasmic reticulum, and even nuclei [6]. Functional studies have shown that MtCK can create permanent contacts between the mitochondrial membranes of reconstituted inner and outer membranes, forming mitochondrial contact sites [10]. Because the nervous system is an organ with a high level of energy consumption, the function of MtCK in neurons is particularly important. Dysfunction of MtCK in the nervous system could lead to serious outcomes. A previous report confirmed that loss of uMtCK is associated with anomalous hippocampal mossy fiber connections, delayed seizure development, among other serious effects [11].

Prior studies have also found that the MtCK activities are decreased in neurodegenerative diseases. One study found that uMtCK activity is approximately 63% lower in HD human brain samples as compared to non-diseased controls [12]. This decrease in MtCK activity could result in a great reduction in the efficiency of energy transfer, and thus lead to neuronal dysfunction. In looking specifically for any changes in MtCK in PD, one study reported a significant decrease in the level of MtCK in cells and animals exposed to dopamine [13]. Considering this, it is likely that there could be changes in the activity of MtCK in the serum of patients with PD. However, we have yet to find a study that explicitly focuses on the study of MtCK in clinical PD patients, and only several studies have investigated the serum CK levels of PD patients [14] and the CK-B activity of PD, idiopathic epilepsy, multiple sclerosis patients, etc.[15]

With this in mind, we sought to investigate the activity of MtCK in the serum of PD patients and controls with the hope that this would allow us to identify a potential biomarker for early diagnosis or progression of PD patients, as well as provide some avenues for further research regarding mitochondrial dysfunction in idiopathic PD.

## METHODS AND MATERIALS

### Patients and ethics statement

This study was approved by the Ethics Committee of Nanfang Hospital. All patients who were enrolled provided written informed consent prior to the start of the study. If informed consent from the patient was impossible, the written informed consent was obtained from the patient’s primary caregiver. A total of 50 PD patients were enrolled from the Nanfang Hospital of Southern Medical University and Guangdong 999 Brain Hospital. Two experienced neurologists assessed each patient and gave a PD diagnosis according to the MDS clinical diagnostic criteria for PD [16]. Additionally, a total of 30 controls were recruited from the outpatient and inpatient populations of Nanfang Hospital during the same period by an experienced neurologist who was blind to the research objectives, according to the inclusion and exclusion criteria that were predetermined. Most of the patients in this group presented with mild headache, dizziness, or other minor neurological deficits. Patients in both the PD and control groups were of Han ethnicity and were excluded from the study for any one of the following reasons: currently taking medication that could influence blood biochemistry with the exception of anti-Parkinsonism drugs, recent or chronic glucocorticoid drug use, myopathy or apparent muscle atrophy, high blood pressure, diabetes, acute inflammatory response, autoimmune disease, any neuroleptic malignant syndrome, as well as the presence of any other severe neurological diseases. All controls included in this study did not have PD, extrapyramidal disorder, any neuromuscular disease, recent trauma of any kind, myocardial infarction, hypothyroidism, any diagnosed psychiatric disease, olfactory disorder, constipation, sleep disorder, or any cognitive impairments.

### Nervous system assessment and neuropsychological testing

The MDS-Unified PD Rating Scale [17], PD Sleep Scale [18] and the Hoehn & Yahr Scale were used to assess all PD patients (ON state) for motor disability and disease severity. We defined five common non-motor symptoms as the Main Non-Motor Symptoms: hyposmia, constipation, neuropsychiatric disorders (depression, anxiety), sleep disturbances, and cognitive impairment. Hyposmia and constipation were assessed by the NMSS [19]. Depression and anxiety were assessed by the Hamilton Depression Scale (HAMD scale) or the Hamilton Anxiety Scale (HAMA scale), respectively [20]. Sleep disturbances were assessed by PD Sleep Scale [18]. Cognitive impairment was assessed by CMMSE [21]. Any main non-motor symptom would be assigned a score of one. The sum of each patient’s main non-motor symptoms was called the Main Non-Motor Symptom Score (MNMSS). An experienced neurologist who had professional training in use of the rating scales and was blind to the research objectives performed all assessments.

Within the PD group, patients were subdivided according to their modified Hoehn & Yahr Scale results into either an early-stage PD group (EP, H&Y stage 1-2.5) or a late-stage PD group (LP, H&Y stage 3-5) [22]. We operationally defined H&Y stage/disease duration as the rate of disease progression. The onset of PD referred to the first time when the patients experienced certain motor symptoms such as bradykinesia, typical tremor, rigidity, etc. Control patients were age- and gender-matched and were divided into an early age-matched control group (hereafter referred to as the “early control” group, or EC) and a late age-matched control group (hereafter referred to as the “late control” group, or LC).

### Sample preparation and storage

A phlebotomist collected blood samples from patients after 8 h of fasting and refrain from any excessive exercise for the three days prior. Samples were then stored in a serum separating gel tube. After coagulation in a 4°C refrigerator, samples were centrifuged at 1500 g for 10 min at 4°C. The top serum layers were immediately collected and stored at -80°C until further analysis.

### Reagents

Both anti-CKMT2 [3F4-G5-H5] (ab131179, anti-sMtCK monoclonal antibody) and anti-creatine kinase MT [1A6-C7-G10] (ab131188, anti-uMtCK monoclonal antibody) antibodies were obtained from Abcam (China) (Shanghai, China). A separate, commercially available kit (CH0101211, lot 0617021) purchased from Maccura Biotechnology (Chengdu, Sichuan, China) was used to determine serum CK-MB activities.

**Table 1:** The demographic & clinical data of the groups studied.

	PD	Control
N	50	30
Age (years)	61.7±8.5	59.8±7.0
Male	21 (42%)	14 (46.7%)
Female	29 (58%)	16 (53.3%)
BMI (Kg/m^2^)	21.1±1.8	20.7±1.4
H&Y stage	2.5±0.8	-
Disease duration (years)	4.9±3.6	-
Age of Onset (years)	56.8±7.9	-
Rate of disease progression	1.0±1.0	-
MDS-UPDRS Part III	32.0±21.2	-
MNMSS	2.9±1.3	0
Tremor dominant (%)	9 (18%)	-
Akinetic-rigid dominant (%)	22 (44%)	-
Mixed subtype (%)	19 (38%)	-
Constipation	32 (64%)	0%
Hyposmia	2 9(58%)	0%
Cognitive impariment	8 (16%)	0%
Depression and/or anxiety	38 (76%)	0%
Sleep disturbances	36 (72%)	0%

Abbreviation: BMI= Body mass index. H&Y stage= modified Hoehn & Yahr stage. Rate of disease progression= Hoehn & Yahr stage/disease duration. MDS-UPDRS Part III= MDS-Unified Parkinson’s Disease Rating Scale (Part III Motor Examination). MNMSS= Main Non-Motor Sympotom Score.

### Instruments

A Beckman AU5431 biochemical auto-analyzer (Beckman Coulter, CA, USA) was used to conduct the immunoinhibition assay for CK-MB serum activities. All of the assays were performed in accordance with the corresponding instrument’s protocol.

### Sample measurements

**Figure 1.**
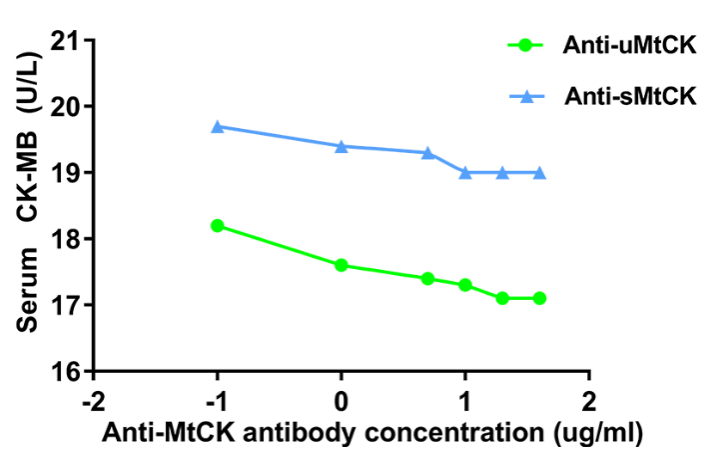
sMtCK and uMtCK activities are shown using anti-sMtCK and anti-uMtCK antibodies, respectively Key: Log transformation was performed on concentration (X axis) of actual dose (-2=lg 0.01, -1=lg 0. 1, 0=lg 1, 1=lg 10, 2=lg 100). A preliminary study confirmed 20 µg/ml was the optimal concentration of the anti-MtCK antibody for serum measurements. uMtCK = ubiquitous Mitochondrial Creatine Kinase, sMtCK = sarcomeric Mitochondrial Creatine Kinase.

We measured the serum MtCK activity according to a previously reported method [23]. Conventional assay of CK-MB activity is an immunoinhibition method against the MM fraction of creatine kinase (CK-M). In the present study, the serum uMtCK activity was the difference between the tested CK-MB values using the conventional CK-MB activity assay and the tested CK-MB values using the anti-uMtCK monoclonal antibodies in addition to the anti-CK-M antibody. The serum sMtCK activity was the difference between the tested CK-MB values using the conventional CK-MB activity assay and the tested CK-MB values using the anti-sMtCK monoclonal antibodies in addition to the anti-CK-M antibody. Prior to actual measurements, we tested a range of primary antibody concentrations for both the anti-sMtCK and the anti-uMtCK antibodies in order to determine the most effective inhibitory concentration. Based on those results, we selected 20 µg/ml as the anti-MtCK antibody concentration for serum measurements (Fig. 1).

**Table 2:** The serum uMtCK, sMtCK, CK-MB activity, BMI in different groups.

Group		N	Age	Serum uMtCK	Serum sMtCK	Serum CKMB
				(U/L)	(U/L)	(U/L)
PD	Total	50	61.7±8.5	3.5±1.8	0.32±0.50	10.2±6.7
	Male	21	62.5±8.8	3.3±2.1	0.32±0.49	10.9±6.3
	Female	29	61.1±8.3	3.6±1.6	0.32±0.53	9.7±7.0
	Early PD	27	59.4±7.0	3.9±1.9	0.28±0.46	10.4±7.3
	Late PD	23	64.3±9.3	3.0±1.5	0.38±0.56	10.1±6.0
Controls	Total	30	59.8±7.0	5.9±1.8	0.34±0.48	9.6±5.5
	Male	14	61.7±6.5	6.5±1.8	0.34±0.48	10.2±6.2
	Female	16	58.2±7.2	5.4±1.7	0.34±0.50	9.1±4.9
	Early control	16	57.6±7.3	5.5±1.6	0.31±0.42	9.7±5.5
	Late control	14	62.4±5.8	6.4±2.0	0.38±0.57	9.5±5.6
p-value (age adjusted)					
	PD vs. controls		0.32	<0.01**	0.73	0.16
	M:F (PD)		0.58	0.54	0.86	0.85
	M:F (C)		0.17	0.16	0.99	0.63
	EP:LP		0.09	0.29	1.00	1.00
	EP:EC		0.42	<0.01**	0.73	0.91
	LP:LC		0.50	**<**0.01**	0.89	0.82
PD subtype	TD	9	60.9±4.8	3.3±1.6		
	AR	22	61.7±9.2	3.5±1.8		
	Mixed subtype	19	62.0±9.2	3.5±2.0		
p-value (age adjusted)		0.95	0.27		
Drug used	With Medopa	44	62.4±8.7	3.5±1.8		
	Without Medopa	6	56.5±3.6	3.4±2.3		
p-value (age adjusted)		0.11	0.91		

Abbreviation: early PD (EP) = early stage PD group. late PD (LP) = late stage PD group. early controls (EC) = early age-matched controls. late controls (LC) = late age-matched controls. TD = tremor dominant. AR = akinetic-rigid dominant.

Key: Bold, significant difference between groups.

### Statistical Analyses

All continuous variables, including uMtCK, sMtCK, and CK-MB activities, as well as age and clinical scale data were expressed as means ± standard deviations. Categorical variables were expressed as frequency, by percent. The data for serum uMtCK, age, as well as BMI were normally distributed, whereas the data for sMtCK, CK-MB, and MNMSS were abnormally distributed. Group mean values were compared using one-way ANOVA if the data were normally distributed with homogeneity of variance. The General Linear Model was used for age and gender adjustment or both. In all other cases, a nonparametric test was used (Mann-Whitney U test or Kruskal-Wallis test). A partial correlation coefficient was used to evaluate the relationships between the different clinical parameters. Linear regression was used to test the relationship between serum uMtCK activities and the disease progression rate, duration, age at disease onset, H&Y stage, and MNMSS. Models were adjusted for age and gender. The receiver operative characteristic (ROC) analysis was conducted to evaluate the diagnostic performance of potential biomarkers. Statistical significance was defined by a p-value of less than 0.05, identified with *. When the p-value was less than 0.01, data are identified with **. All data were analyzed using the standard statistical package SPSS v. 19.0 (IBM, Chicago, IL, USA) and graphical depictions were made using Graphpad Prism v. 7.03 (GraphPad Software, Inc. La Jolla, CA, USA).

## RESULTS

### Demographic and clinical data of enrolled patients

All recorded demographic and clinical parameters for both the enrolled PD and control patients are listed in Tables 1 and 2. The number of patients in the two groups was comparable. No significant differences were identified in patient age. All controls were lacking any non-motor symptoms, such as: constipation, cognitive impairment, depression and anxiety. The akinetic-rigid dominant subtype accounted for the largest proportion of all PD patients.

**Figure 2.**
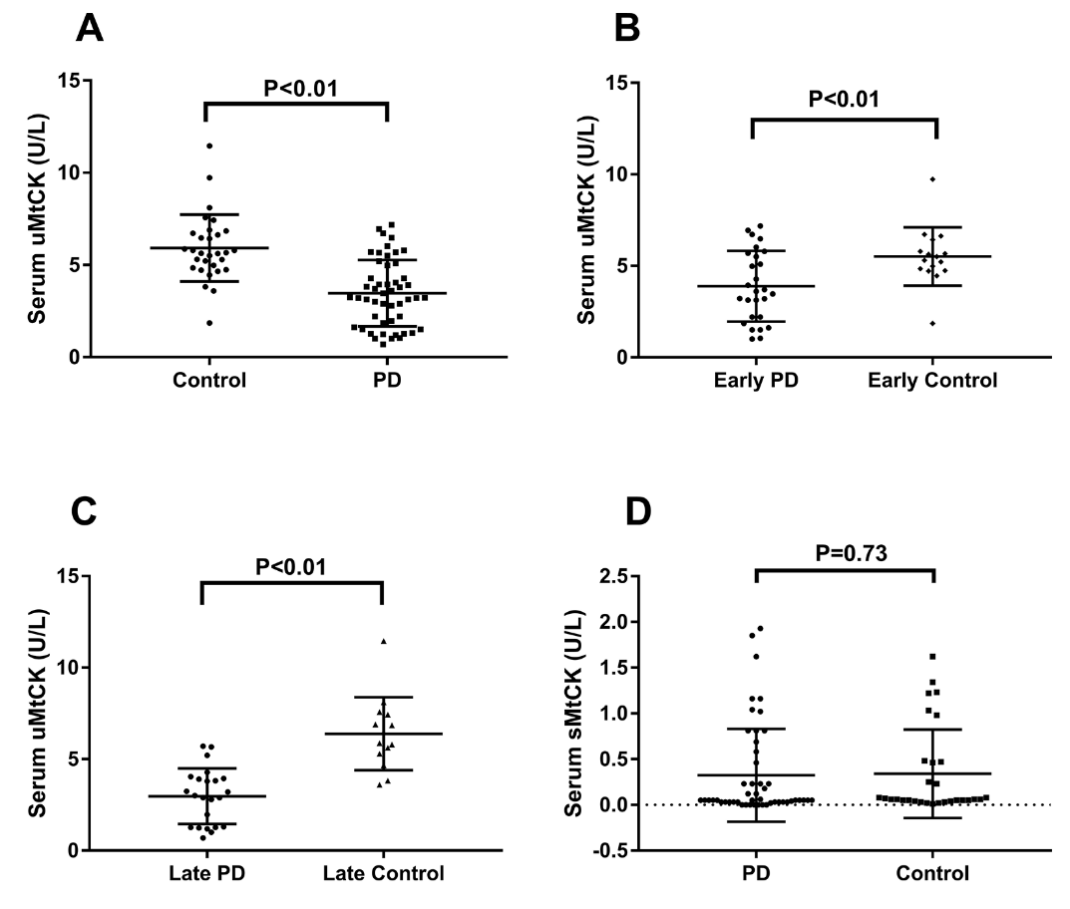
Comparisons of serum activities of uMtCK and sMtCK between the different patient groups **A**) The PD group had a significantly lower serum uMtCK activity when compared with controls (p<0.01). **B, C**) Stratified analysis was performed according to disease severity (H&Y stage), revealing a significant decrease in serum uMtCK activity between the EP and EC groups (p<0.01) as well as the LP and LC groups (p<0.01). **D**) No significant difference in serum sMtCK activity was observed between the PD and control groups (p=0.73).

**Figure 3.**
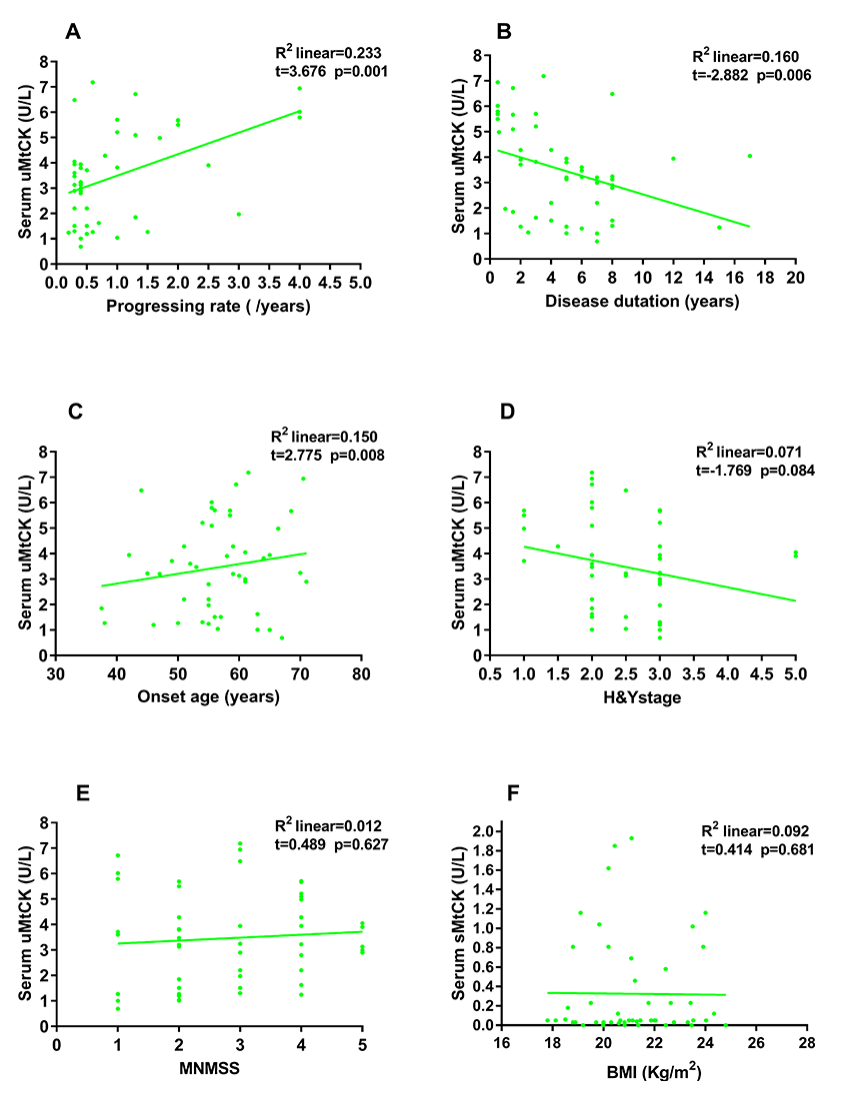
Correlation between serum uMtCK activities and rate of disease progression, disease duration, age of onset, H&Y stage, and MNMSS in PD patients Serum uMtCK activities significantly increased along with the accelerating rate of disease progression (R^2^=0.233, t=3.676, p=0.001), as well as with older age of onset (R^2^=0.150, t=2.775, p=0.008), but significantly decreased along with extended disease duration (R^2^=0.160, t=-2.882, p=0.006) (**A-C**). No significant correlation was observed between serum uMtCK activities and either H&Y stage or MNMSS (**D, E**) or between serum sMtCK activities and BMI (**F**).

### Comparison of uMtCK, sMtCK, and CK-MB serum activities between PD and control groups

The PD group exhibited significant decrease in serum uMtCK activities compared with control group. We also found a significant difference between the LP and the LC groups as well as the EP and the EC groups (Table 2, Fig. 2). There was no significant difference between the LP and the EP groups, although serum uMtCK activities for the former were lower than the latter. Serum sMtCK and CK-MB activities were not significantly different between any of the aforementioned groups. Stratified analysis by gender showed there was no significant difference between male and female PD patients in terms of serum levels of uMtCK, sMtCK, or CK-MB activity, which was also true for the control group (Table 2). All p-values were adjusted for age.

### Comparison of serum uMtCK activities among the different clinical dominant types of PD patients.

We found no significant difference in different clinical dominant types (tremor dominant, akinetic-rigid dominant, and mixed subtype) of PD patients, although the cases for the tremor dominant group were relatively small (Table 2).

### Correlation between serum uMtCK activities and rate of disease progression, disease duration, age of onset, H&Y stage, and MNMSS, *and between* serum sMtCK activities and BMI

To explore the underlying relationships between the changes in serum uMtCK activities that were observed, we next conducted both correlational and linear regression analyses with other disease-relevant parameters. We found a statistically significant, positive correlation between serum uMtCK activity and the rate of disease progression and age of onset. We also found a significant, inverse correlation between serum uMtCK activities and disease duration (Fig. 3 A-C). However, there were no significant correlations between serum uMtCK activities and H&Y stage, MNMSS, nor were there any between serum sMtCK activities and BMI (Fig. 3 D-F). All p-values were adjusted for both age and gender.

### Diagnostic value of serum uMtCK activity in PD patients

**Figure 4.**
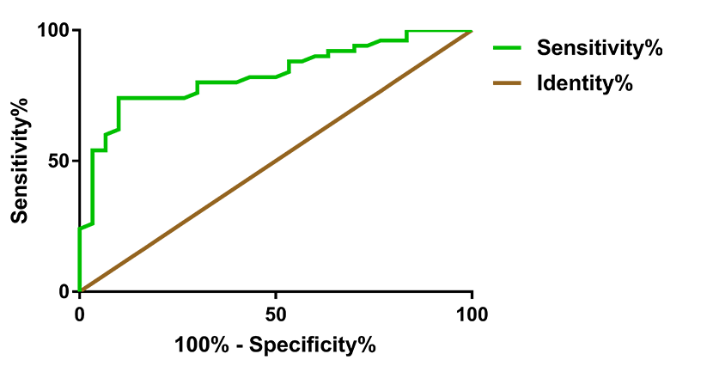
ROC curve of serum uMtCK activity The area under the curve (AUC) was 0.83 (P<0.01) (95% CI: 0.74 - 0.92). When the uMtCK activity was less than 4.37 U/L, Youden’s index was maximal with a sensitivity of 74.00% (95% CI: 59.66-85.37%) and a specificity of 90.00% (95% CI: 73.47-97.89%). ROC= receiver operator characteristic.

Receiver operating characteristics analysis indicated that a cutoff activity of 4.37 U/L resulted in a sensitivity of 74.00% (95% CI: 59.66-85.37%) and a specificity of 90.00% (95% CI: 73.47-97.89%), with an area under the curve of 0.83 in order to discriminate PD from control subjects (P<0.01) (Fig. 4).

## DISCUSSION

The importance of mitochondrial function in the production of energy through the mitochondrial respiratory chain (RC) goes without saying, and is obviously critical to many cellular processes, including the regulation of cell death, calcium metabolism, and the generation of reactive oxygen species (ROS) [24]. There have been many studies on mitochondrial dysfunction as it relates to PD [2-4]. Some authors have concluded that mitochondrial dysfunction, especially respiratory chain damage, is a major cause of idiopathic PD [25]. It is well known that MtCK is involved in the transmission of energy and is responsible for transferring the energy produced by mitochondria to the cytoplasm. There are two subtypes of MtCK: uMtCK and sMtCK. However, the status of MtCK in the body fluids of patients with PD is unknown. Therefore, our study sought to explore any differences in the MtCK activities, uMtCK and sMtCK, in the serum of PD patients. Our results showed that the serum uMtCK activity was significantly lower in PD patients when compared with controls. Further statistical analysis showed that the decline of uMtCK in the serum of PD patients was associated with the rate of disease progression, the disease duration, and the age of onset.

There are several reasons that can explain this decline in uMtCK activity. First, it may be an adaptation to both the decrease in mitochondrial function and the decrease in energy supply observed in PD patients [4]. Acting as an energy buffer system, MtCK might adapt to the new pathological state presented by PD, thereby reducing its expression. Another proof of this phenomenon was the observation that serum MtCK activities were significantly higher in patients with hepatocellular carcinoma (HCC) than in those without HCC or healthy subjects, a result of a higher metabolic state [26]. Second, it is also likely that PD patients would have a significantly reduced number of mitochondria due to severe mitochondrial damage, which would subsequently lead to a remarkable decrease in uMtCK secretion into the peripheral blood. Although currently we do not have direct histological evidence to support our hypothesis, it has been previously confirmed in separate pathological studies that PD patients do exhibit decreased mitochondrial protein levels [27-29]. To determine whether or not motor symptoms could affect the activity of uMtCK, we analyzed them in different dominant subtypes. Our results indicated that there were no significant differences in the serum uMtCK activity among the three different subgroups, suggesting that these motor symptoms might not affect uMtCK activity.

Correlational analyses showed that the decline in uMtCK activity in the serum was related to both the rate of progression and duration of disease. Here, we operationally defined the disease progression rate as H&Y stage (number) /disease duration (years). Using this definition, we found that serum uMtCK activities were positively correlated with the rate of disease progression in PD patients. Our data also indicated a link between serum uMtCK activity and both disease duration and age of onset. This could mean that the younger the patient’s age at onset of disease, the longer the disease duration, and thus the more obvious mitochondrial dysfunction. Although close to establishing a significant relationship with H&Y stage, our serum uMtCK activity data fell short of statistical significance. This could be due to our relatively small sample size. The number of patients determined to be in H&Y stages IV and V was relatively small, further reducing our statistical power. What’s more, we could not find a correlation between serum uMtCK activities and UPDRS-III score, which could be disturbed by different therapeutic drug effects among patients.

In addition to motor symptoms, non-motor symptoms also occur in PD patients. For instance, past work has shown that the staging of brain pathology is related to sporadic PD [30], indicating that new symptoms manifest with progression of disease. On this basis, we also investigated the main non-motor symptoms (MNMSS) in our patient population. Unfortunately, we failed to find a correlation between serum uMtCK activities and MNMSS in this study. This could be due to the relatively large number of non-motor symptoms shown in our patient population. Another possible reason for the lack of significant correlation between uMtCK and motor symptoms or non-motor symptoms is that when motor symptoms are present, many neurons, including dopaminergic neurons in the substantia nigra and sympathetic nerves in the heart and gastrointestinal tract, have been lost [31], leaving limited room for uMtCK activities to drop. Our results also indicated that uMtCK activities in patients with late PD were lower than those in patients with early PD, however, this finding was not statistically significant.

Contrastingly, serum sMtCK activities were not significantly different between the PD and control groups. This was interesting given that both sMtCK and uMtCK are major types of MtCK [10, 32]. We think that the main reason for this is the different locations of these different subtypes. More specifically, sMtCK is predominantly found in the mitochondria of myofibrillar sarcomeres in striated muscle tissue, whereas uMtCK is found in mitochondria of a wide variety of other tissue cells, mainly in the nervous system [6]. The sequestered location of sMtCK may result in reduced sensitivity to general body changes as compared to uMtCK. For example, several oncology studies have shown that patients with a poorer prognosis exhibit uMtCK overexpression [33]. Moreover, our study showed that serum CK-MB activities were not significantly different between the PD group and the control group. Although it is an important part of the energy transfer system, CK-MB is mainly located in the cytoplasm of cardiomyocytes, so it is not easily affected by mitochondrial dysfunction in PD. Previous research has supported our conclusion on this aspect [15]. Interestingly, Hideki Takubo found increased creatine kinase in PD patients, compared with controls. The authors explained that the elevated creatine kinase may be related to sensitivity to physical exercise and neural mechanism mediated by hypothalamic dopamine or by autonomic nervous system[14]. In combination with our results, we speculated that the increase of CK may be also related to mitochondrial dysfunction. When mitochondrial dysfunction, ATP production decreased. CK in the cytoplasm might have compensatory responses, showing the increase in activity, trying to obtain more ATP.

There are several factors that could affect creatine kinase activity, two of which are drug treatment [14] (*e.g.* levodopa) and muscle atrophy or weight change. In our study, the drug Medopa did not notably affect serum uMtCK activities. Another manifestation of PD is weight change [34, 35], and taking this into consideration, we also analyzed the relationship between BMI and sMtCK activities. However, we found no significant correlation. It appeared that, despite the possible presence of weight change in patients with PD, there was no significant effect on serum sMtCK. We postulate that a potential reason for this is that PD is a highly heterogeneous disease, and that weight changes would be varied, possibly increasing, decreasing, or remaining unchanged [36].

It should be noted that our study had some limitations. First, the sample size was relatively small, which may have influenced our statistical power and, subsequently, our results. Future research should use a larger sample size to counteract these potential effects. Second, we were unable to provide baseline data for our patients, making it difficult to evaluate dynamic changes to each PD patient’s MtCK activity. Third, in order to offer further insight into the etiopathogenesis of idiopathic PD, future work will need to study the specific mechanism behind changes to uMtCK. Finally, we must further investigate the activity of uMtCK in the CSF and closely examine any correlation that exists between serum and CSF uMtCK activity.

### Conclusion

In summary, our study showed that the uMtCK activity in the serum of PD patients was significantly lower than in the controls. However, examination of serum sMtCK activity among the same groups did not result in any significant differences. We hypothesized that the different locations of these kinases accounted for these different results. Additionally, we found a positive correlation between serum uMtCK activity and both the rate of disease progression and age at time of onset. Contrastingly, serum uMtCK activity was inversely correlated with disease duration. Overall, our study provides a critical and helpful clue for more in-depth research into potential biomarkers for mitochondrial dysfunction in PD.
